# The cost-effective, but forgotten, medical endometriosis therapy: a prospective, quasi-randomized study on progestin therapy

**Published:** 2018-12

**Authors:** TC Cezar, KW Schweppe, KR Pletzer, S Becker, H Krentel, LA Torres-De La Roche, RL De Wilde

**Affiliations:** Gynecology Consultant. Scientific Researcher at the University Hospital for Gynecology, Pius Hospital, University Medicine Oldenburg, Carl von Ossietzky University, Oldenburg, Germany. Email: christina.cezar@uni-oldenburg.de;; Professor of the Clinic of Gynecology and Obstetrics at Ammerland – Klinik Westerstede, Westerstede, Germany, Schweppe@Ammerland-Klinik.de;; Gynecology Consultant at Clinic for Gynecology and Obstetrics of Ammerland – Klinik Westerstede, Westerstede, Germany, cristina.cezar@msn.com;; Professor and Director of the Clinic of Gynecology and Obstetrics Universitätsklinikum Frankfurt, Frankfurt, Germany, sven.becker@kgu.de;; Chief physician of the Clinic of Gynecology, Obstetrics and Oncology, St. Anna Hospital, Herne, Germany, gynaekologie@annahospital.de;; Gynecology Consultant. Scientific Researcher at the University Hospital for Gynecology, Pius Hospital, University Medicine Oldenburg, Carl von Ossietzky University, Oldenburg, Germany. E-mail: luzangela.torres-delaroche@pius-hospital.de;; Professor and Head of the University Hospital for Gynecology, Pius Hospital, University Medicine Oldenburg, Carl von Ossietzky University, Oldenburg, Germany. Email: rudy-leon.dewilde@pius-hospital.de

**Keywords:** cost-effective, endometriosis, progestin, low-cost medication, compliance

## Abstract

Endometriosis represents nowadays a real burden for the patients as well as for the physicians, as it requires surgical and/or medical treatment, often long – termed and repeated. Moreover, the high costs necessary to diagnose and treat endometriosis represent a real economic burden, being comparable to other chronic diseases like diabetes or rheumatoid arthritis. Therefore, the physicians dealing with this disease should take into account not only the efficacy of the treatment, but also the economic aspects and patients compliance.

The present paper analyses the efficiency of progestins (lynestrenol and medrogestone) in endometriosis as a cost – effective, but forgotten medical therapy of the disease. Our study underlines the good tolerability of progestins, as they have limited side effects, the compliance of patients being high. They are also low-cost medications, which could represent an effective alternative method in the endometriosis treatment, especially in less – developed countries that cannot afford the higher therapeutic costs.

## Introduction

Endometriosis is a chronic recurrent disorder, caused by the presence and proliferation of endometrial glands and stroma outside the uterine cavity (Valle and Sciarra, 2003; Gylfason et al., 2010). It represents a real burden for women’s quality of life, being a chronical disease which requires surgical (Healey et al., 2010) and/or medical treatment, often long - termed and repeated (Nnoaham et al., 2011; Vercellini et al., 2014) . Furthermore, it is associated with substantial costs as the economic burden of endometriosis is high and comparable to other chronic diseases like diabetes or rheumatoid arthritis (Levy et al., 2011; Simoens et al., 2012). Thus, the goals of the treatment should take into consideration, not only the efficacy aspects, but also issues like products costs and patients compliance (Simoens et al., 2012) .

In the management of endometriosis, the principal objective is relieving the symptoms, especially the pain (Chwalisz et al., 2002; Elnashar, 2015; Jensen, et al., 2018). The secondary endpoint of therapy is to prevent recurrence or to approach a delay of complaints of the disease through surgical methods or medically-induced atrophy of endometriosis implants (Rice, 2002; Valle and Sciarra, 2003). Surgical treatment remains an important step in the management of this disease (Ulrich et al., 2014), but as long as endometriosis is hormonally sensitive (Burney and Giudice, 2012), the combined medical and surgical treatment continues to be standard (De Wilde et al., 2018). Among medical treatments, progestins have been successfully used in the therapy of endometriosis for more than 40 years (Schweppe, 2001). Due to their central and peripheral action, progestins inhibit the estrogen-induced proliferation and mitogenic mechanism (Li et al., 2011; Zito et al., 2014). Their direct effects on endometrium create a pseudopregnancy – state, due to increased decidualization and atrophy of both eutopic endometrium, as well as of endometriotic lesions (Schweppe, 200; ESHRE, 2001). All these mechanisms of action, together with reduced menstrual flow, manage to improve the symptoms of endometriosis during therapy with progestins (ESHRE, 2001; Vercellini, et al., 2003a; Bayoglu Tekin et al., 2011; Kim, et al., 2013).

The main goal of the present paper is to bring up in a new light the forgotten medical endometriosis therapy through progestins, in an era dominated by expensive or low compliance endometriosis medications, exhausting and demanding surgical procedures for the patients as well as for the physicians. In order to strengthen the theoretical aspects of this issue, we applied the results of an old prospective quasi randomized study, which were saved in the database of the university clinic Oldenburg as a doctor title manuscript document. In this respect the effectiveness of progestins was studied, through parameters like improvement of subjective complaints of the patients, amelioration of clinical, laparoscopic and histological examination as much as the patients compliance considered by different side effects and easiness of the therapy intake.

## Materials and methods

In this prospective, quasi – randomized study, lynestrenol and medrogeston were compared in a group of 47 patients, suffering from endometriosis, histologically confirmed. The study was performed between 1985 and 1989 and it was designed as quasi – experimental, being planned pre – interventional, without ethical committee agreement and registered at German Clinical Trials with the registration number DRKS0017159. At that time only informed consent of the patients was required as the three – stage therapy of endometriosis with intermediate progestin (first – look laparoscopy – progestin therapy – second – look laparoscopy) was considered standard (Mettler & Semm, 1983). In all the cases, a first – and second – look laparoscopy was performed and the suspected areas were histologically investigated.

The patients were quasi – randomized in two groups:

- L – group, including the patients born on odd days, who received 10 mg/p.o./die lynestrenol

- M – group, with patients born on even days, whom were treated with 10 mg/p.o/die medrogeston.

In case of uterine bleeding during the study period, the dosage of progestins was doubled or further increased.

The therapeutic period varied between 4.17 and 9.07 months, depending on the subjective symptoms and stage of endometriosis, possible side effects of the progestins’ therapy, which sometimes required to further increase the dose of medication. The patients were re – examined at an interval of three months and following parameters investigated: subjective complaints, clinical physical examination, laboratory tests and side effects (Table I, II, III). At the end of progestin therapy, a second – look laparoscopy was performed in all cases and the remaining endometriosis lesions and scars were excised.

**Table I:** Classification of endometriosis – related symptoms

Dysmenorrhea	Dyspareunia	Dysuria	Painful bowel movements
0 – none	0 – none	0 – none	0 – none
1 – little influence of wellbeing, no analgesics required	1 – discomfort during intercourse	1 – dependent on menstrual cycle, dragging sensation by micturition	1 – occasionally constipation, dependent on menstrual cycle and/or discomfort by defecation
2 – analgesics sporadically required	2 – often painful intercouse	2 – dependent on menstrual cycle, painful micturition	2 – frequently constipation, dependent on menstrual cycle and/or discomfort by defecation
3 – analgesics several days required	3 – avoidance of very painful intercouse	3 – disorders of micturition, due to the pain	3 – disorders of defecation, due to the pain

**Table II:** Clinical findings by bimanual palpation

Abdominal pain	Uterus	Pain/tension	Nodular induration and tumoral change
0 – none	0 – normal, anteversio, anteflexio, normal consistency and mobility	0 – none	0 – none
1 – minor impairment of well - being, no analgesics required	1 – retroflexio uteri, mobile	1 – Douglas and/or adnexal pain	1 – nodular induration in Douglas
2 – occasionally analgesics required	2 – anteflexio, fixed		2 – adnexal mass, unilaterally
3 – frequently analgesics required	3 – retroflexio, fixed		3 – adnexal mass, bilaterally

**Table III:** Side effects intensity of lynestrenol and medrogeston therapy

0 – none
1 – no influence of subjective well being
2 – minor influence of subjective well being
3 – major influence of subjective well being

## Results

The average age of the patients was 27.6 years, ranging between 19 and 40 years. 16 patients of the L-group addressed our clinic because unfulfilled family planning, 15 of them having a primary subfertility and 1 of them being diagnosed with secondary subfertility. In the M – group sterility was observed in 10 patients (8 primary and 2 secondary sterility).

In L-group, 4 patients had had a missed abortion, 5 had already given birth to one child and 19 women had never been pregnant before therapy. In M-group, 4 women had had a missed abortion and two patients had one child and two children, respectively. 14 women were nulligravida. Menstrual irregularity with a cycle longer than 30 days was seen in 3 cases of L-group and 2 patients of M-group.

In 24 patients of the L-group, the endometriosis was primarily diagnosed, whereas 3 women had a previous laparoscopic diagnose of the disease. In the M-group, there was only one patient with recurrent disease, who previously received danazol (Table IV). The main patients’ complaints are emphasized in table V.

**Table IV:** Patients’ demographics in L-group and M-group

Patients’ demographics	Term	L – group (n = 27)	M – group (n = 20)
Nulligravida	n (%)	19 (70%)	14 (70%)
Nulliparous	n (%)	22 (81%)	17 (85%)
Age of menarche	x mean ± 1 SD (years)	12,63 ± 1,09	13,25 ± 1,37
Menstrual cycle	x mean ± 1 SD (days) min. max.	28,48 ± 6,5 33,93 ± 14,78	27,25 ± 4,59 31,30 ± 4,56
Duration of bleeding	x mean ± 1 SD (days) min. max.	3,78 ± 1,26 5,04 ± 1,17	3,85 ± 0,79 5,10 ± 0,89
Gyn OP’s (laparoscopy or laparotomy)	n (%)	7 (26%)	8 (40%)
Known Endometriosis	n (%)	3 (11%)	1 (5%)
Contraception	n (%)	3 (11%)	5 (25%)

**Table V:** Clinical symptoms of the patients in L-group and M – group.

Symptom	Intensity	Start of therapy		Middle of therapy		End of the therapy		After the therapy	
		L	M	L	M	L	M	L	M
Abdominal pain	0	33,33	50	48,15	55	77,77	65	61,90	77,77
	1	0	5	44,44	35	18,52	15	28,57	11,11
	2	51,85	35	7,41	10	3,70	15	4,76	11,11
	3	14,82	10	0	0	0	5	4,76	0
Dysmenorrhea	0	70,73	70	96,30	90	100	95	76,19	100
	1	7,41	15	3,70	5	0	5	19,05	0
	2	14,81	10	0	5	0	0	0	0
	3	7,41	5	0	0	0	0	4,76	0
Dyspareunia	0	70,73	75	81,48	80	92,59	80	90,48	100
	1	29,63	10	18,52	20	7,41	15	4,76	0
	2	11,11	15	0	0	0	5	4,76	0
	3	0	0	0	0	0	0	0	0
Painful defecation	0	100	95	92,59	90	92,59	85	95,24	0
	1	0	5	7,41	10	7,41	15	4,76	0
	2	0	0	0	0	0	0	0	0
	3	0	0	0	0	0	0	0	0

In both groups, the most common symptom at the beginning of therapy was abdominal pain, followed by dysmenorrhea, dyspareunia and dyschezia.

In L-group, at the beginning of therapy 18 patients (66.67%) had abdominal pain of different intensity, out of which 14 (51.85%) claimed to still have pain in the middle of treatment and 6 of them (22.22%) also at the end of therapy. However, the intensity of pain decreased in all cases. The rate of dysmenorrhea decreased from 29.63% (8 patients) to 0%, after approximately 6 months of treatment. Dyspareunia was present in 8 patients (29.63%) and decreased to a rate of 18.52% (5 cases) in the middle of therapy and to 7.41% (2 patients) at the end of therapy. Only 2 patients (7.41%) claimed to have mild painful defecation.

In the M-group, abdominal pain of different intensity was present in 10 cases (50%). Out of these cases, 9 (45%) still had pain at the examination under treatment and 7 (35%) at the end of therapy. Dyspareunia was observed in 5 patients (25%) at the beginning and in 4 patients (20%) in the middle and at the end of therapy. The rate of dysmenorrhea fell from 30% (6 patients) at the beginning to 5% (1 case) at the end of therapy. Painful defecation was present in 5% (1 patient) at the beginning and in 15% (3 cases) at the end of therapy.

After ending the therapy, patients were followed up for a period of 9,9 months (1-24 months) in L-group and of 9.6 months (1-21 months) in M-group. In the L-group, out of 21 patients who came for follow up, 8 (38.1%) still claimed to have abdominal pain, however of a lower intensity than pretherapeutic. 5 Cases (23.81%) still claimed to have dysmenorrhea. In M-group, 9 patients were followed up and 2 of them (38.1%) had recurrent abdominal pain. Other symptoms were not mentioned.

### Clinical examination

Abdominal pain and tenderness in adnexal and Douglas region were decreased in clinical examination of patients in both groups (Table VI). Palpable, nodular induration and tumorous masses clearly improved under therapy with medrogeston.

**Table VI:** Bimanual palpation findings in L-group and M-group (%).

	Intensity	Start of therapy		End of the therapy	
		L	M	L	M
Pain	0	55,56	65	88,89	90
Tension	1	44,44	35	11,11	10
Nodular induration and tumoral mass	0	66,67	75	81,48	90
	1	7,41	0	0	0
	2	25,93	25	11,11	10
	3	0	0	7,41	0
Uterus position and mobilization	0	70,73	75	77,78	84,21
	1	18,52	10	18,52	15,79
	2	7,41	10	0	0
	3	3,70	5	3,70	0

In 2 patients from L-group, bilateral adnexal tumors were palpable at the end of therapy. One of them had no adnexal tumor before therapy and the other one had an adnexal mass on the left side before starting lynestrenol.

In the L-group, at the beginning of therapy, uterus position and mobilization was normal in 19 patients (70.37%), whereas 4 cases (18.52%) had a retroflexio uteri mobilis and one case had a retroflexio uteri fixata. In the latter case, the same clinical situation was still noted at the end of therapy. In 2 cases (7.41%), the uterus was previously fixed and after therapy regained its normal mobility.

In the M-group, uterus position and motility was normal in 75% of patients. There were 2 cases with retroflexio uteri mobilis and one case with retroflexio uteri fixata. In the latter patient, the uterus could be normally mobilized at the end of therapy.

### Side effects of progestins

Different side effects of the therapy are summarized, for both groups, in Table VII.

**Table VII:** Side effects of therapy.

s	L – group (n = 27)	M – group (n = 20)
Spotting	20	17
Headache	1	0
Depression	1	2
Fatigue	1	1
Edema	3	0
Nausea/vomiting	0	3
Breast tenderness	1	0
Decreased libido	1	0

One of the most common side effects was uterine bleeding, usually appearing as “spotting”, which was seen in the first months of treatment in 20 patients (74.07%) of L-group. In most of these cases, the bleeding was resolved by increasing the dose to 30 mg. At the end of therapy, there were still 9 cases (33.3%) with bleeding of mild intensity. There was only one patient with persistent bleeding which required an increased dose of 100 mg.

In L-group, there were 3 cases with significant bilateral leg swelling. All of these patients also complained of headache, depressive mood, fatigue, breast tenderness and reduced libido. These symptoms slightly altered the general condition of health, but were completely reversible after the end of therapy.

The uterine bleedings were also seen in 17 cases of M-group (85%) and were resolved after increasing the dose to 30-40 mg. There was only one case in which an increased dose of 90 mg was necessary to stop the bleeding. At the end of therapy, there were only 7 cases with spotting. Three patients claimed to have nausea and vomiting under therapy. A depressive mood was present in two patients and one of them prematurely ended the study, after 4,5 months because of this.

In 20 patients of L-group (74.07%), a weight gain of 2.59 kg was reported, ranging from 0.6 to 8.5 kg, over a period of 3 months (Table VIII). At the end of therapy, there were 24 patients (88,89%) with an average weight gain of 3.68 kg, ranging between 0 and 9 kg. In M-group, weight gain was noted in 13 patients (65%) in the middle of therapy and in 15 patients (75%) at the end of therapy. In this group, the average weight gain was 0.97 kg (-5 to 5 kg) in the middle and 2.43 kg (-1 to 9 kg) at the end of therapy.

**Table VIII:** Weight gain in the different groups.

s	Start of therapy		End of the therapy	
	L	M	L	M
Weight gain (in %)				
0-1 kg	37,04	65	25,93	50
1-3 kg	25,93	25	29,63	20
3-5 kg	29,63	10	11,11	20
>5 kg	7,41	0	33,33	10
Mild weight gain (kg)	2,59	0,97	3,68	2,43
Standard deviation ± 1 SD	2,36	2,08	2,68	2,65
Minimal weight gain (kg)	0	-5	0	-1
Maximal weight gain (kg)	8,5	5	9	9

### Laparoscopic examination

In both groups, the most frequent localization of disease was in the pouch of Douglas, followed by the sacrouterine ligaments deep and superficial (Table IX). Ovarian location was on the third place, being observed in 7 patients. Bilateral ovarian endometriosis was present in 2 L-group cases and one M-group case. Large endometriomas were present in 4 patients of each group. Tubal endometriosis associated with different adhesions was very rarely documented.

**Table IX:** Location of endometriosis masses.

Location	L – group (n = 27)	M – group (n = 20)
Uterus	1	0
Vesical peritoneum	2	3
Douglas	19	16
Sacrouterine ligaments	11	10
Ligg. rotunda	1	0
Ligg. lata	2	4
One ovary	7	7
Both ovaries	2	1
One tube	3	4
Both tubes	6	7
Cervix uteri	0	1

In order to define therapeutic success in both groups, the endometriosis classification of AFS (American Fertility Society) was used (Table X).

**Table X:** Laparoscopic endometriosis findings after AFS classification (%).

Stage	Points	L – group Start of therapy	L – group End of therapy	M – group Start of therapy	M – group End of therapy
0	0	0	29,63	0	15,79
I	1 – 5	51,85	48,15	40	36,84
II	6 – 15	22,22	18,52	40	31,58
III	16 – 40	25,93	3,70	15	15,79
IV	> 40	0	0	5	0
x mean		10,41	3,81	9,85	8,11
± SD points		11.43	6,76	10,74	9,60

In L-group, a clear recovery of the disease was observed. At the begin of therapy, there were 7 patients (25.93%) in stage III and at laparoscopic control, this stage was diagnosed only in one case (3.7%). Disease improved to stage I in 2 cases and to stage II in 4 cases. 6 Patients (22.22%) of L-group were laparoscopically diagnosed with endometriosis stage II. In 2 of these cases, there was no endometrial mass observed at the end of therapy and 4 of them had an improvement of disease to stage I. Out of 14 patients (51.85%) with stage I at the first diagnostics, 6 had normal gynecological findings, 6 patients still had a stage I disease and in one patient a failure was recorded with upgrading to stage II, at the end of therapy.

In one M-group patient (5%), endometriosis changed macroscopically from stage IV to stage III at the end of therapy. In three cases (15%), there was an endometriosis stage III diagnosed at begin of therapy. After therapy with medrogeston, the stage of disease improved to level II in one patient whereas the other 2 cases remained unchanged. Out of 8 patients (40%) with a stage II, there were 4 cases with improved to stage I, and 3 cases unchanged. In one case, second look laparoscopy was not performed, because of intercurrent pregnancy.

Eight patients were diagnosed at the beginning with endometriosis stage I. Three of them, in each group had a normal gynecological examination at the end of therapy. In the other two patients, there was a stage II diagnosed.

### Histological examination at the beginning of therapy

There were 21 L-group cases and 16 M-group cases, in which the endometriosis biopsy was made in proliferative phase. Out of these, there were 7 (33.3%) cases in L-group and 3 (18.75%) cases in M – group low differentiated, without signs of functional modulation. Out of the biopsies made in secretory phase (6 in L-group and 4 in M-group), there were 2 (33.3%) in L-group and 1 (25%) in M – group low differentiated. There were mostly cystic dilated glands, with row cubical or segmental cylindrical epithelium observed.

The histological examinations at the beginning of therapy recorded following differentiating grades: in L-group (n=27), there were 9 cases (33.3%) low differentiated; 6 specimens (22.22%) mixed differentiated and 12 cases (44.4%) highly differentiated.

In the M-group, there were 4 specimens (20%) diagnosed as undifferentiated. In 7 cases (35%) there was a mixed differentiation and in 9 biopsies (45%) a high differentiation was reported.

Close to the endometriosis areas, bleeding of different intensity was observed, partially also macrophages with deposits of hemosiderin.

### Histological examination at the end of therapy

At the second – look laparoscopy after 6 months of therapy, with lynestrenol, n=27, respectively with medrogeston, n=19, there were no signs of endometriosis at all, in 9 L-group patients (33.3%) and in 3 M-group cases (15.8%). Therefore, there were only 18 histological specimens in L-group and 16 in M-group. In one patient of M-group, no second look laparoscopy was performed, because of pregnancy.

Out of 18 specimens in L-group, there were 7 cases without any active glands. In these situations, only fibrotic changes of stroma were noticed: a complete healing of the disease was considered. In 11 cases, despite 6 months lynestrenol therapy, the endometriosis persisted. However, in 8 biopsies (44.4%) atrophic, non – functional glands and stroma were observed. In 3 specimens (16.6%) endometriosis were active and proliferative.

Out of 16 specimens of M-group, there were 6 (37.5%) biopsies without any signs of active endometriosis glands. In 4 cases (25%) there were regressive, nonfunctional glands with flattened epithelium. Only 6 cases (37.5%) showed an active, proliferative endometriosis.

### Pregnancy rate

Out of 16 L-group patients, who addressed our clinic for unfulfilled family planning, 3 became pregnant 2, 3 and 4 months after ending the therapy. All of them had a normal pregnancy course.

Out of 11 symptomatic patients of L-group, one patient became pregnant 8 months after ending the therapy and delivered through cesarean section. After 21 months, she was pregnant again and gave birth to her second child per vaginam. Therefore, the pregnancy rate in L-group was 18.75% after therapy, during the first 12 months.

In M-group, one patient with unexplained subfertility became pregnant 3 months after ending the therapy. She gave birth to a healthy child. Therefore, the pregnancy rate in M-group was 10%.

## Discussion

Our study was conducted between 1985 and 1987, at that time, only informed consent of patients was needed, as the three stage therapy with intermediate progestins was considered standard of care (Mettler & Semm, 1983).

Among patients with endometriosis, the incidence of endometriosis – associated subfertility varies between 30 and 40% (Fritz and Speroff, 2011; Somigliana, et al., 2015). However the rates in this study were higher, 59.26% of L-group subjects and 50% of M-group patients suffering from primary or secondary subfertility.

One year after therapy with lynestrenol, 3 patients with primary or secondary subfertility were pregnant, representing a rate of 18.75% in L-group (n=16). The spermiograms of the partners were not taken into consideration. In the M-group, one patient of those with sterility (n=19) was pregnant 3 months after therapy. This patient had an irregular treatment with medrogeston, having paused the therapy sometimes. Therefore, this pregnancy could not be considered a result of an efficient therapy with medrogeston. From this point of view, lynestrenol proved to be more effective than medrogeston.

Pelvic pain and infertility represent the major clinical problem for the patients (Garry, 2004; Fauconnier and Chapron, 2005; Stratton and Berkley, 2011; Berlanda et al., 2013), in this study being reported in 40.7% of L-group patients and 50% of M-group patients. However, no direct correlation was observed between the intensity of symptoms and laparoscopic findings. A similar lack of direct relationship between the stage of endometriosis and the intensity of symptoms claimed by the patients is documented in the literature (Fauconnier, et al., 2002; Vercellini, et al., 2007).

At begin of therapy, 18 L-group patients (66.6%) had pain of different magnitude, at the middle of therapy 14 had pains (51.85%) and at the end of treatment there were still 6 patients (22.2%) with this symptom. However, in all cases, the intensity was clearly decreased.

In the M-group, 10 women (50%) claimed to have pain of different intensity, at the beginning. Out of these cases, in the middle of treatment, 9 patients still had pain (45%). At the end of therapy, the symptom was still present in 7 patients (35%).

The rate of painful defecation increased under therapy to 7.41% (L-group) and 10% (M-group). A possible explanation of this phenomenon could be the action of progestin on intestinal muscles: reducing the intestinal tonus of smooth muscles. From this point of view, lynestrenol had a better effect compared to medrogeston.

In regard to clinical examination, in L-group, the painful symptoms were reduced with 33.33% and the palpable induration decreased with 14.82%. The pain was reduced in M-group with 25% and the induration with 15%.

Regarding the side effects reported to medication, there were no differences observed to the data published (Berlanda et al., 2016). The most frequent adverse events noted were bleeding and weight gain. The bleeding could be resolved in most of the cases by increasing the dose of medication.

However there was a moderate tendency to a more frequent and greater weight gain in the lynestrenol group. Other side effects noted in the series were leg edema (3 cases in L-group), nausea and vomiting (3 patients of M-group). Further adverse reactions were seen in both groups only in isolated cases and didn’t influence the general condition of patients. The frequency of side effects was similar to the literature, reported by different authors (Schweppe, 2001; Kennedy, et al., 2005; Schweppe, 2012). All adverse events disappeared completely after therapy.

A second - look laparoscopy was performed after approximately 6 months, in order to resect the remaining endometriosis. In the L-group, an improvement of disease from 10.41 points (±1.43) at the beginning of therapy to 3.81 points (±6.76) at the end of treatment was observed. In M-group, these data were 9.85 points (±10.74) to 8.11 points (9.60). Therefore, there was an improvement in L-group from a middle stage II to a stage I and in M-group remained by a middle stage II. A complete healing of the disease was recorded in 8 L-group cases (29.63%) and in 3 M-group cases (15%). Therefore, lynestrenol proved to be significantly superior to medrogeston.

Regarding the histological regression of endometriosis, the results obtained with lynestrenol were better than those in medrogeston group. According to the data published in literature (Schweppe, 2001; Li, et al., 2011; Brown, et al., 2012), our histological findings demonstrated a clear progression from undifferentiated, cystic dilated ectopic endometrial foci to high differentiated spots, similar to normal endometrium, although partially several stages of differentiations were seen in some biopsies.

As emphasized in the present study, the use of progestins in endometriosis treatment has several important advantages, which should be taken into consideration by clinicians who are dealing with this disease.

The use of progestins after surgical removal of endometriosis is useful in relieving the pain symptoms (Brown, et al., 2012) and delaying recurrence (Vercellini, et al., 2003a; Abou-Setta, et al., 2013).

Given the fact that endometriosis possibly requires a long medical treatment, irrespective of performed surgical excision, being a chronic and recurrent disease (Vercellini, et al., 2003a; Krentel, et al., 2017), the therapy with progestins could represent an efficient alternative method, especially in less – developed countries, which cannot tackle the higher costs of novel medical treatment. The tolerability is good, having limited metabolic effects and have a convenient application, being inexpensive and easily to use (ESHRE, 2001; Vercellini et al., 2003a).

## Conclusion

In conclusion, our study emphasizes the good tolerability of progestins, as they have limited side effects, in contrast to other agents. The compliance of patients is high, due to reduced side effects and easy usage. Furthermore, they are low – cost medications, which is an important aspect to be taken into consideration, as progestins could represent an effective alternative method to treat endometriosis, especially in less – developed countries, that cannot afford the higher costs of treatment.

In this study lynestrenol – showed superior to medrogestone – therapy and should be reconsidered as an effective, safe, long – to – use and low – cost progestin therapy in endometriosis.
